# Image dataset: Optimizing growth of nonembryogenic citrus tissue cultures using response surface methodology

**DOI:** 10.1016/j.dib.2024.111091

**Published:** 2024-10-30

**Authors:** Randall P. Niedz, Eldridge T. Wynn

**Affiliations:** Agricultural Research Service, U.S. Horticultural Research Laboratory, 2001 South Rock Road, Ft. Pierce, FL, USA

**Keywords:** Plant tissue culture, Design of experiments, Callus, Sweet orange, In vitro, Image analysis

## Abstract

The data are images of Valencia sweet orange nonembryogenic tissue grown on different culture media that varied in the composition of the mineral nutrients from three experiments. Experiment 1 was a 5-factor d-optimal response surface design of five groupings of the component salts that make up Murashige and Skoog (MS) basal salt medium. Experiment 2 was a 3-factor d-optimal response surface design of extended ranges of factors 1, 2, and 3 from Experiment 1. Experiment 3 was thirteen formulations that were predicted using the prediction model generated from the 5-factor RSM from Experiment 1. The predictions were for two types of growth. One, points were predicted where growth was equal to MS medium (the standard), and two, points predicted with growth greater than MS medium by a minimum of 25%. An image representative of each formulation in each of the experiments makes up the dataset. The data will be useful for 1) visualizing the effects of the diverse mineral nutrient compositions, effects that may not be fully captured with single measure metrics; 2) development of image analysis applications via computer vision and segmentation algorithms for additional insight or for more rapid and possibly accurate assessment of tissue growth and quality; and 3) as an educational resource to learn how to use multifactor experimental designs to assess in vitro growth.

Specifications Table

**Table:** 

Subject	Horticulture
Specific subject area	In vitro growth of nonembryogenic sweet orange callus tissue.
Type of data	Raw, Image
Data collection	The images were captured with a Nikon Coolpix 5400 digital camera at a resolution of 2592 × 1944 pixels. Culture plates containing tissue were photographed in a laminar flow hood illuminated with cool white, fluorescent lights, with the top cover of the culture dish removed. One culture plate representative of each treatment was photographed.
Data source location	Data collected at the USDA Horticultural Research Laboratory, Ft Pierce, FL (27.42795845982308, -80.40889238507944) Data stored at the United States, USDA National Agricultural Library (39.022807354728855, -76.92179156871161).
Data accessibility	Repository name: USDA Ag Data Commons Direct URL to 5-factor RSM data: https://doi.org/10.15482/USDA.ADC/26514073 Direct URL to 3-factor RSM data: https://doi.org/10.15482/USDA.ADC/26663131 Direct URL to 5-factor RSM prediction data: https://doi.org/10.15482/USDA.ADC/26665372
Related research article	Niedz, R.P., Evens, T.J. Regulating plant tissue growth by mineral nutrition. In Vitro Cell. Dev. Biol. Plant 43, 370–381 (2007). https://doi.org/10.1007/s11627-007-9062-5

## 1 Value of the Data

• Visualization of the results. The images document what the tissue looked like for each treatment, information not fully captured by conventional single measures such as color, fresh and dry weights, and friability. This would be useful for researchers studying mineral nutrition of in vitro plant cultures as well as students learning plant tissue culture.
• Image analysis applications. The images in combination with the experimental design structures can be used to develop applications for image segmentation and tissue classification suitable for generating quantitative measures for analysis. Such measures could compliment or possibly even replace some of the conventional measures.
• Teaching design of experiments. The images were taken at the points specified for a design of experiments (DOE) response surface design.

## 2 Background

Plant tissue culture is the in vitro culture of plant cells, tissues, and organs and is used for commercial and research applications. What applications can be used for a particular plant species and genotype are dependent on the availability of tissue culture systems that can grow the cells and tissues as they need to be grown. The study was motivated to determine if a multivariate approach would be useful for improving growth compared to the standard MS medium, the most widely used plant tissue culture medium. The literature on developing tissue culture formulations is dominated by comparing single culture formulations/recipes. In contrast, this experiment was structured as a multivariate response surface to quantify the main, interaction, and quadratic effects of the mineral nutrient salts used in MS medium, and to use the resulting model to improve growth. A relatively easy-to-grow cell line of the Valencia sweet orange, a widely grown commercial orange, was used. The image data compliments the fresh weight data by visually capturing what the cultures looked like at the different design points (formulations) in what was a 5-dimensional design space and should be useful for image analysis and developing image analysis algorithms and applications.

## 3 Data Description

The data are images of tissue cultures of Valencia sweet orange nonembryogenic callus cells. The objective of the project was to determine the effects of mineral nutrition on callus growth using response surface methodology (RSM) [1]. The dataset includes three experiments documented as follows –

1. Experiment 1 – A 5-factor RSM design. The design matrix is presented in the spreadsheet file **5 factor RSM Design_11-28-2005.xlsx**. The design included 46 runs divided into 3 blocks. Six culture dishes were used to estimate the response for each run. A culture dish representative of the run was photographed. Each image is named to match the run of the design. For example, the jpg image labeled **Run 04_Blk1_5F RSM_NE_Valencia_11-28-2005.JPG** is an image of the callus grown on Run #4 that was part of Block 1 of the 5-factor RSM design for the nonembryogenic Valencia sweet orange callus cells. This dataset includes 47 files – 46 image files and 1 Excel spreadsheet [2].
2. Experiment 2 – A 3-factor RSM design. The design matrix is presented in the spreadsheet file **3 factor RSM Design_7-1-2005.xlsx**. The design included 20 runs. Six culture dishes were used to estimate the response for each run. A culture dish representative of the run was photographed. Each image is named to match the run of the design. For example, the jpg image labeled **Run 11_3F RSM_NE_Valencia_7-1-2005.JPG** is an image of the callus grown on Run #11 that was part of the 3-factor RSM design for the nonembryogenic Valencia sweet orange callus cells. This dataset includes 22 files – 21 image files and 1 Excel spreadsheet [3].
3. Experiment 3 – Thirteen points (formulations) that were predicted using the prediction model generated from the 5-factor RSM from Experiment 1. These points and their factor levels are listed in the spreadsheet file **5 factor RSM Design_Predicted.xlsx**. The purpose was to determine how well the prediction model from Experiment 1 predicted growth of the callus. The predictions were for two types of growth. One, points were predicted where growth was equal to MS medium (the standard), and two, points predicted with growth greater than MS medium by a minimum of 25%. These two growth conditions are labeled as ‘Equal to MS growth’ and ‘25% > MS growth’ in the spreadsheet. ‘Equal to MS growth’ includes 7 predicted points, ‘25% > MS growth’ includes 6 predicted points, and a point for the MS control. Six culture dishes were used to estimate the response for each point. A culture dish representative of growth at that point was photographed. Each image is named to match the point. A couple of examples – the jpg image labeled **5-factor RSM_MS prediction 10.JPG** is an image of the callus grown on point #10 predicted by the 5-factor RSM model to have growth equivalent to MS, and the jpg image labeled **5-factor RSM_MS+25% prediction 16.JPG** is an image of the callus grown on point #16 predicted by the 5-factor RSM model to have growth at least 25% greater than MS. This dataset includes 15 files – 14 image files and 1 Excel spreadsheet [4].

## 4 Experimental Design, Materials and Methods

The experimental design utilized a 5-factor D-optimal response surface approach, suitable for modeling a quadratic polynomial. This design was enhanced by adding ten extra points for lack-of-fit analysis. A total of eleven points were replicated to estimate pure error, which included one specific point that was repeated twice for the MS medium. The number of replicates was determined based on the minimum degrees of freedom required for a statistically valid estimation of pure error. The design points chosen in both experiments, including the replicate points, were selected to ensure a statistical power greater than 90%. Each treatment involved six duplicate plates, meaning that each replicate included an additional six plates.

Tissue samples of approximately 1 gram were placed on growth medium in culture dishes measuring 100 mm in diameter and 20 mm in height. The cultures were maintained for a 14-day period in a controlled environment chamber. The chamber conditions included low-intensity illumination (15-20 µmol.m^-2^.s^-1^) provided by cool-white fluorescent lighting, a constant temperature of 27°C, and a short 4-hour photoperiod. The cultures underwent two rounds of subculturing during this time. At the beginning of the third growth cycle (day 0), the culture plates were weighed both with and without the tissue samples. The initial tissue mass was calculated by subtracting the empty plate weight from the total weight of the plate with tissue. This process was repeated on day 14 to determine the final tissue mass. Prior to the final weight measurement, photographs of the tissue cultures were taken under cool-white, fluorescent lighting to document their appearance.

**Fig. 1:**
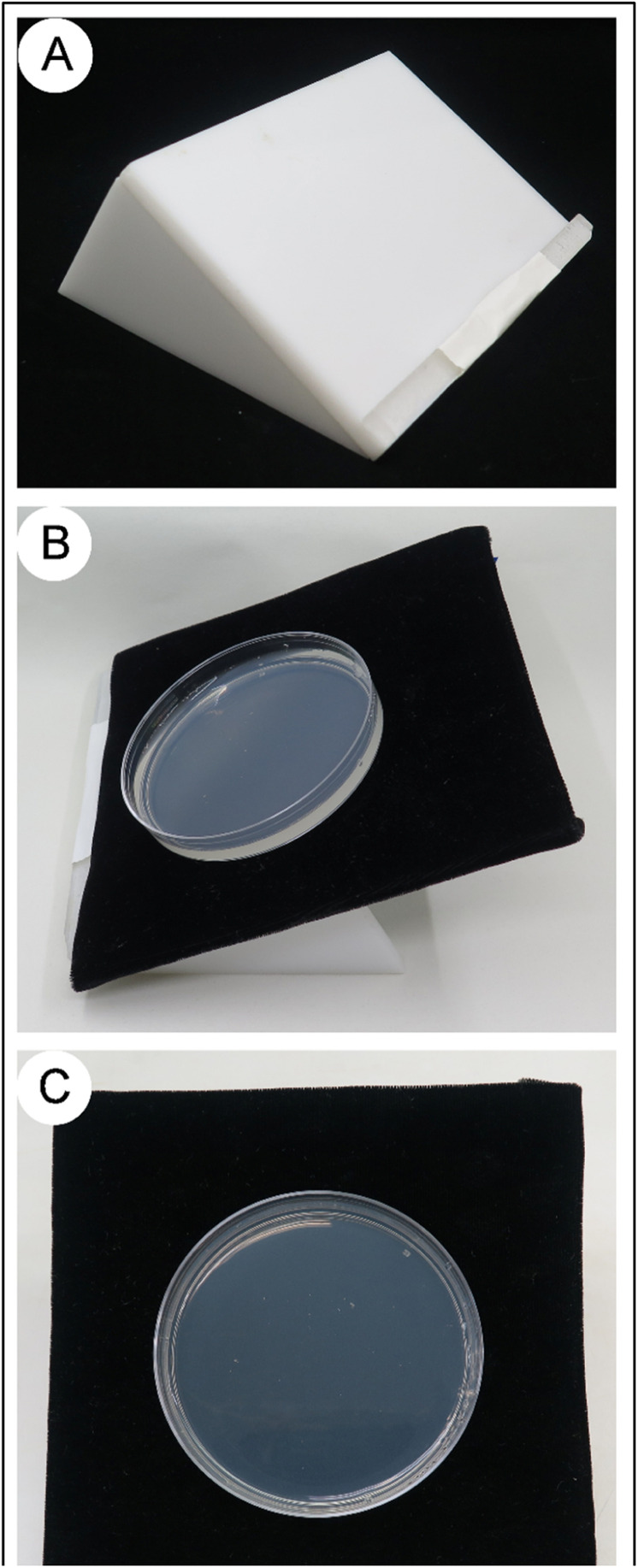
Acrylic stand and black velvet background to support culture dish for photographing. A) Acrylic stand. B) Acrylic stand, black velvet background, and petri dish setup for photography. C) Perspective of the view to take photographs.

Images were captured in JPEG (EXIF 2.2) format with a Nikon Coolpix 5400 digital camera equipped with a 1/1.8” (7.2 × 5.3 mm) CCD sensor at a resolution of 2592 × 1944 pixels. Each image is of a single culture plate with the top lid removed. The culture plate was placed on a black velvet background on an angled acrylic support stand (Fig. 1). The acrylic support was constructed from 6 mm thick acrylic sheets cut and glued with an acrylic cement. The sides of the stand consisted of two pieces cut into right triangles (5 cm × 9 cm × 10 cm) with angles of 30^o^, 60^o^ and 90^o^. A rectangular piece (14 cm × 9.5 cm) was attached to the side pieces allowing the plates to be presented at a 30^o^ angle. A slice of clear acrylic sheet (14 cm × 8.5 mm × 6 mm) was attached to the rectangular piece to provide a ledge for petri plates or the backdrop. A 15 cm × 15 cm sheet of Bio-Rad Extra Thick Block Filter Paper #1703959 (Bio-Rad Laboratories, Hercules, California, USA) was covered with black velvet, placed on the stand and used as the backdrop for photos to provide contrast.

## Limitations

A limitation of these image data sets is that the image for each formulation is the most representative culture dish for that formulation. Though this accurately captures what was observed, it does not capture the variation of each formulation. For each formulation six culture dishes were used. For the conventional measures, such as fresh weight, the average of the six cultures was used to estimate the response of that formulation. In contrast, the plate that was closest to the average response was used for the image. Another possible limitation was the JPG format used. JPG uses lossy compression, which means some image data is discarded to reduce file size, and because the data is compressed artefacts can be introduced. A better format would be TIFF format because it uses lossless compression and therefore preserves all image data.

## Ethics Statement

1. The authors have read and followed the ethical requirements for publication in Data in Brief and confirm that the current work does not involve human subjects, animal experiments, or any data collected from social media platforms.
2. The images of this work have not been published previously.
3. The manuscript is not under consideration for publication elsewhere.
4. Each author has approved the manuscript for publication, along with the responsible authorities where the work was carried out, either tacitly or explicitly.
5. If accepted, the article will not be published elsewhere in the same form, whether it's in English, another language, or electronically without the copyright-holder's consent.

## CRediT Author Statement

**Randall Niedz:** Conceptualization, Methodology, Writing- Original draft preparation, Writing- Reviewing and Editing, Data Curation. **Eldridge Wynn:** Methodology, Writing- Reviewing and Editing.

## Data Availability

Ag Data CommonsImage data of growth of Valencia sweet orange nonembryogenic cells on points from a 3-factor response surface design to determine the effects of mineral nutrition on growth. (Original data). Ag Data CommonsImage data of growth of Valencia sweet orange nonembryogenic cells on predicted points from a 5-factor response surface design. (Original data). Ag Data CommonsImage data of growth of Valencia sweet orange nonembryogenic cells on points from a 5-factor response surface design to determine the effects of mineral nutrition on growth. (Original data).
